# *Abiotrophia defectiva* endophthalmitis following routine cataract surgery: the first reported case in the United Kingdom

**DOI:** 10.1099/acmi.0.000124

**Published:** 2020-03-23

**Authors:** Madalina Chihaia, James Richardson-May, Layth Al-Saffar, Hiron Kettledas, Mohammed Rashid

**Affiliations:** ^1^​ Trust Grade Registrar, Ophthalmology, Royal Bournemouth Hospital, Dorset, Bournemouth, UK; ^2^​ Specialty Registrar Year 2, Ophthalmology, Royal Bournemouth Hospital, Dorset, Bournemouth, UK; ^3^​ Consultant Microbiologist, Ophthalmology, Royal Bournemouth Hospital, Bournemouth, UK; ^4^​ Consultant Ophthalmologist, Dorset County Hospital, Dorchester, UK; ^5^​ Consultant ophthalmologist, Royal Bournemouth Hospital, Bournemouth, UK

**Keywords:** *Abiotrophia defectiva*, endophthalmitis, cataract, ophthalmology, eye

## Abstract

**Introduction:**

*
Abiotrophia defectiva* is a fastidious organism that has been implicated in severe infections such as endocarditis in immunocompetent patients. Modern tools are available to aid identification, but the main challenge remains clinical suspicion of *A. defectiva *.

**Case presentation:**

An otherwise fit and well 65-year-old female presented with reduced vision, red eye and discomfort 2 days following routine left cataract surgery. She had visual acuity of light perception only, significant anterior chamber inflammation (including hypopyon) and limited fundal view. She was diagnosed with post-operative endophthalmitis and 0.1 ml of ceftazidime (2 mg/0.1 ml) and 0.1 ml vancomycin (2 mg/0.1 ml) were injected intravitreally after vitreous aspiration. Subconjunctival cefuroxime was also injected. A repeat injection was performed on day three of admission. Gram staining revealed Gram-positive long-chain cocci, which were identified as *A. defectiva *. The patient was discharged on oral ciprofloxacin 500 mg twice a day with oral prednisolone 60 mg once a day; this was tapered and stopped at 8 weeks post-discharge. The left eye received dexamethasone 0.1 % 6 times a day (again, tapered over 8 weeks), moxifloxacin 5 % 6 times a day and atropine 1 % twice a day. Vision improved to 6/12 unaided (6/9.5 with pinhole) at 9 weeks post-operatively, with a clear fundal view.

**Conclusion:**

We present a case of *A. defectiva* endophthalmitis following routine cataract surgery. To our knowledge, this is the first reported case in the UK and the fourth globally, which with prompt treatment ended with a good visual outcome.

## Introduction

*Abiotrophia defectiva* is a fastidious micro-organism, classified as a Gram-positive, non-motile, facultative aerobe, which was separated from the genus *Streptococcus* in 1995 after genetic and phylogenetic analyses by 16S rRNA gene sequencing [1, 2]. *Abiotrophia* strains are differentiated from other *Streptococci* by nutrient requirements, satellitism and pyrrolidonyl arylamidase activity [3]. Part of the normal human microbiota, this organism inhabits the oral, genitourinary and intestinal mucosae [4]. It is not usually found in the conjunctival sac or ocular adnexae [5].

Difficult to cultivate, it is highly virulent and can affect endovascular structures. It has subsequently been identified as the cause of numerous culture-negative endocarditis cases, including in immunocompetent patients [6], some with recent history of dental treatment [7]. *A. defectiva* is associated with septic embolization, but isolated *de novo* peripheral infections have also occurred. Cases of sepsis, osteomyelitis, brain abscess, pancreatic abscess and septic arthritis have also been reported [8].

*A. defectiva* is able to produce exopolysaccharides and adheres to the fibronectin in the extracellular matrix, offering a mechanism for its virulence [9]. The frequent colonization and infection of cardiac valves suggest an affinity for avascular tissue [10]. It has also been isolated from dental plaques and intra-articular prostheses, which could indicate binding properties to acellular or synthetic materials [3, 11].

In ophthalmology, the literature presents cases of crystalline keratopathy [12], infiltrative keratitis in contact lens wearers [13] and post-keratoplasty keratitis with hallmark features of well-circumscribed infiltrates and intense inflammatory reactions [5, 14]. Post-operative endophthalmitis following cataract surgery has been described, twice in routine cases [15]. One of these demonstrated retinal involvement and acute infiltrative keratitis post-operatively [16]. Further, *A. defectiva* endophthalmitis has occurred following cataract removal with posterior capsule rupture and anterior vitrectomy [17]; as secondary to a dexamethasone-implant [18]; and bleb-associated [3].

Being difficult to isolate and requiring a special medium with supplemental sulfhydryl compounds (e.g. 1-cysteine) and pyridoxal to grow, the role of *Abiotrophia* organisms as an causative agent might be underestimated [2]. New advances in its identification have been made through the introduction of alternative methods, such as automatic phenotypic tests using biochemical substrates (VITEK 2, Biomerieux, France), matrix-assisted laser desorption/ionization time-of-flight mass spectrometry (MALDI-TOF MS; Bruker Daltonik, Germany) and 16S rRNA gene sequencing by polymerase chain reaction (PCR) [2, 19].

## Case report

### Presentation

A routine cataract extraction (via phacoemulsification) and intraocular lens (IOL) implantation were performed under general anaesthesia in the left eye of a 65-year-old female who was otherwise fit and well, with no recent dental procedures. She had previously undergone uncomplicated right phacoemulsification and IOL insertion. Intracameral antibiotic (cefuroxime; Aprokam) was used according to the national guidelines. The intended postoperative treatment was guttae (g.) dexamethasone 0.1 % and g. chloramphenicol 0.5 %, each 4 times a day for 4 weeks. The vision improved immediately following the operation.

Two days later the patient reported reduced vision, mild conjunctival hyperaemia and an ache over the eyebrow in the left eye. She was seen in our hospital out of hours.

The vision in the left eye was perception of light. Clinical examination revealed no relative afferent pupillary reflex (RAPD) bilaterally and a quiet, pseudophakic right eye. The left eye showed mild conjunctival hyperaemia with ciliary flush, a 0.5 mm hypopyon, 3+ cells in the anterior chamber and 2+ flare. There were conglomerates of fibrin around the pupil, limiting fundal view. Also present was 360° of posterior synechiae; this was successfully broken nasally with a stat dose of g. tropicamide 1 % and g. phenylephrine 2.5 %. The intra-ocular pressure (IOP) was 18 mmHg. Ocular B-scan (ultrasound) showed a flat retina and vitreous opacities.

### Diagnosis and treatment

The diagnosis of post-operative pan-endophthalmitis was established and the patient was admitted for a vitreous tap and injection of intravitreal antibiotics, which was performed within 90 min from admission; 0.1 ml ceftazidime (2 mg/0.1 ml) and 0.1 ml vancomycin (2 mg/0.1 ml) were injected. The scleral wound was sutured with 8.0 Vicryl. Subconjunctival cefuroxime was injected at the end of the procedure.

Gram staining of the vitreous sample revealed Gram-positive long-chain cocci. The patient was prescribed oral ciprofloxacin 500 mg twice a day. The left eye received g. dexamethasone 0.1 % 6 times a day, g. moxifloxacin 5 % every hour and g. atropine 1 % twice a day.

The second day after admission, the clinical appearance deteriorated with a 1 mm hypopyon and increased cells in the anterior chamber; this was felt likely to indicate a post-treatment inflammatory reaction. Oral prednisolone 60 mg once a day was initiated.

A second intravitreal antibiotic injection was performed on the third day of admission, on day five post-operatively. The patient was counselled of a guarded prognosis. The microbiology department reported moderate growth of *A. defectiva *, sensitive to ceftriaxone (MIC 1 mg l^−1^). The species was identified after growing on fastidious anaerobic agar, though not on blood or chocolate agar. Direct disc testing also revealed resistance to metronidazole. The MALDI-TOF MS technique (Bruker Daltonik, Germany) gave a good identification of 2.219 (with a score ≥2.3 being ‘highly probable’ for species identification, and≥2.0 but<2.3 suggesting probable identification according to the manufacturer’s instructions).

The patient was discharged the following day with the above treatment, to reduce g. moxifloxacin 5 % to 6 times a day. Follow-up was arranged in the patient’s original hospital, where the cataract surgery was performed. The vision was hand movements and fundal view remained limited.

Over the next 3 months, the clinical appearance improved. The antibiotics (oral and topical) were stopped at 2 weeks following discharge. The oral prednisolone was reduced by 10 mg/week, then by 5 mg/week and was stopped at 8 weeks following discharge. A similar strategy was adopted for the topical dexamethasone 0.1 %.

### Outcome and follow-up

The vision in the left eye reached 6/12 (0.34 LogMAR) unaided, 6/9.5 (0.2 LogMAR) with pinhole at 9 weeks after the cataract extraction. Excluding a mild posterior capsular opacity, the anterior segment was quiet. Fundus view was now possible, though some vitreous condensations persisted. A mild intra-retinal haemorrhage was visible in the inferior quadrant, but otherwise the retina was flat and unremarkable. The optic disc and the macula had a normal appearance.

## Discussion

To our knowledge, this is the first case reporting *A. defectiva* endophthalmitis following cataract surgery in the United Kingdom and the fourth in the literature, with the previous three cases being reported in the USA [15], Spain [17] and Germany [16]. Our case is similar to that described by Namdari *et al*. [15], in which endophthalmitis occurred secondary to a routine phacoemulsification with IOL implantation and a good visual outcome was achieved after intravitreal treatment with ceftazidime and vancomycin, without vitrectomy. Esteban *et al*. [17] reported endophthalmitis following phacoemulsification with posterior capsule rupture and anterior vitrectomy, which is associated with a higher risk of infection. Horstkotte *et al*. [16] described endophthalmitis associated with infiltrative keratitis, which provides a possible entry site for micro-organisms. In addition, it should be noted that it is good practice during cataract surgery to completely remove the viscoelastic material from the capsular bag and from behind the IOL, as it could facilitate bacterial growth in the presence of contamination.

The above-mentioned cases were identified in tertiary centres, which is not surprising, given that modern equipment is required to identify this fastidious organism. *A. defectiva* infection may be underdiagnosed. The pleomorphic appearance of strains may cause diagnostic difficulty, which could lead to prolonged morbidity and costly diagnostic and therapeutic procedures.

The ability of the organism to adhere to fibronectin, which is present at the junction between the vitreous and the retina, may allow the microbe to reside in close proximity to the retinal nerve fibres; this could lead to rapid and irreversible deterioration in vision [14]. The use of core vitrectomy as part of the treatment pathway is debatable. Although it theoretically decreases the micro-organism load in the vitreous cavity, it could risk bacterial spread towards the posterior pole.

Reports in the literature have not found significant levels of resistance among *A. defectiva *. In endophthalmitis cases the key element of the management plan is the primary vitreous tap and intravitreal injection of antibiotics (with current recommendations suggesting vancomycin with ceftazidime). Following identification of the organism, appropriate adjustment of the antimicrobial treatment can occur. The European Society of Cataract and Refractive Surgery (ESCRS) recommends keeping samples of aqueous and vitreous in Eppendorf tubes at −20 °C to perform a PCR subsequently if the cultures are negative [14].

Given previous isolation from endocarditis patients with valve replacements, as well as from patients with intra-articular prostheses, *A. defectiva* may demonstrate adhesion to synthetic materials – including the intraocular lens implant. Interestingly, in our case, the inflammatory reaction in the anterior chamber was not very severe at the initial presentation, with a minor conjunctival hyperaemia. However, significant fibrin deposits were noticed in the pupillary area, raising the suspicion of bacterial adhesion to the IOL.

## Conclusion

Given its pleomorphism and fastidious properties, *A. defectiva* is difficult to isolate, and as such may be underdiagnosed. This has been improved with the introduction of modern identification techniques, but it remains important to consider this organism when presented with culture-negative cases. The therapeutic standard in endophthalmitis cases is intravitreal antibiotic injection after vitreous tap, with appropriate adjustment of therapy should microbiological identification suggest so.
